# The ties that bind the sagebrush biome: integrating genetic connectivity into range-wide conservation of greater sage-grouse

**DOI:** 10.1098/rsos.220437

**Published:** 2023-02-22

**Authors:** Todd B. Cross, Jason D. Tack, David E. Naugle, Michael K. Schwartz, Kevin E. Doherty, Sara J. Oyler-McCance, Ronald D. Pritchert, Bradley C. Fedy

**Affiliations:** ^1^ School of Environment, Resources and Sustainability, University of Waterloo, Waterloo, Ontario, Canada; ^2^ Habitat and Population Evaluation Team, US Fish and Wildlife Service, 32 Campus Drive, Missoula, MT, USA; ^3^ W.A. Franke College of Forestry and Conservation, University of Montana, Missoula, MT, USA; ^4^ USDA Forest Service, National Genomics Center for Wildlife and Fish Conservation, Rocky Mountain Research Station, 800 East Beckwith Avenue, Missoula, MT, USA; ^5^ US Fish and Wildlife Service, Lakewood, CO, USA; ^6^ Fort Collins Science Center, US Geological Survey, Fort Collins, CO, USA; ^7^ Habitat and Population Evaluation Team, US Fish and Wildlife Service, 3425 Miriam Avenue, Bismarck, ND, USA

**Keywords:** conservation prioritization, network model, landscape resistance, functional connectivity, reserve design, spatial action maps

## Abstract

Conserving genetic connectivity is fundamental to species persistence, yet rarely is made actionable into spatial planning for imperilled species. Climate change and habitat degradation have added urgency to embrace connectivity into networks of protected areas. Our two-step process integrates a network model with a functional connectivity model, to identify population centres important to maintaining genetic connectivity then to delineate those pathways most likely to facilitate connectivity thereamong for the greater sage-grouse (*Centrocercus urophasianus*), a species of conservation concern ranging across eleven western US states and into two Canadian provinces. This replicable process yielded spatial action maps, able to be prioritized by importance to maintaining range-wide genetic connectivity. We used these maps to investigate the efficacy of 3.2 million ha designated as priority areas for conservation (PACs) to encompass functional connectivity. We discovered that PACs encompassed 41.1% of cumulative functional connectivity—twice the amount of connectivity as random—and disproportionately encompassed the highest-connectivity landscapes. Comparing spatial action maps to impedances to connectivity such as cultivation and woodland expansion allows both planning for future management and tracking outcomes from past efforts.

## 1. Introduction

Functional connectivity, observed as gene flow among populations, is a fundamental concept poorly integrated into imperilled species conservation [1]. The deterioration of genetic connectivity (i.e. the degree to which gene flow affects evolutionary processes within populations [2]) can lead to isolated populations that are at greater risk of extirpation and to the detrimental effects of small population sizes [3], all of which can often be mitigated by targeted management actions [4,5]. Missing in conservation are the spatial action maps delineating where and how much a given action is likely to contribute to achieving stated goals [6].

The ability to quantify and visualize connectivity has improved rapidly with concomitant advances in genetic sampling and analytical approaches using non-invasive genetic approaches [7]. Contemporary approaches can contrast genetic data with landscape features known to influence individual movement, allowing identification of spatial patterns in genetic connectivity [8]. Furthermore, network analysis can distil complex genetic patterns into a simplified structure of nodes and edges ranked by importance to overall connectivity [9].

Connectivity mapping for conservation is most effective when developed as a two-step process: identifying population centres important to maintaining genetic connectivity, then delineating the pathways most likely to facilitate connectivity. Identifying pathways through habitat-based analyses and prioritizing by pathway benefits to populations enables decision makers to integrate connectivity into conservation designs, though this remains elusive in practice likely due to large-scale sampling requirements.

The greater sage-grouse (*Centrocercus urophasianus*; hereafter, sage-grouse) is a species of conservation concern ranging across eleven western US states and two Canadian provinces. Sage-grouse populations are poised to benefit from the collection of greater than 16 000 genetic samples across their range [8,10–12]. Sage-grouse populations vary substantially in population density and dispersal [13]; these two parameters are fundamentally influenced by connectivity, and both influence the genetic diversity of populations. Populations on the species periphery are at particular risk and have subsequently been the target of any translocation efforts [14–16]. Additionally, peripheral populations can demonstrate negative demographic outcomes including the dampening or loss of cyclicity in population trends [17]. Row *et al*. [13] found that the greatest loss of genetic variation resulted from changes in dispersal (i.e. connectivity) from central to more peripheral populations. Sage-grouse genetic connectivity typically occurs among neighbouring populations [18–23], though factors shaping connectivity of distant populations include terrain and regionally variant land use and land cover changes such as cultivation and woodland expansion [8]. Sage-grouse are capable of dispersing long distances among discrete breeding areas known as leks (less than or equal to 194 km) [24] and among seasonal habitats (less than or equal to 160 km) [25,26].

**Figure 1.**
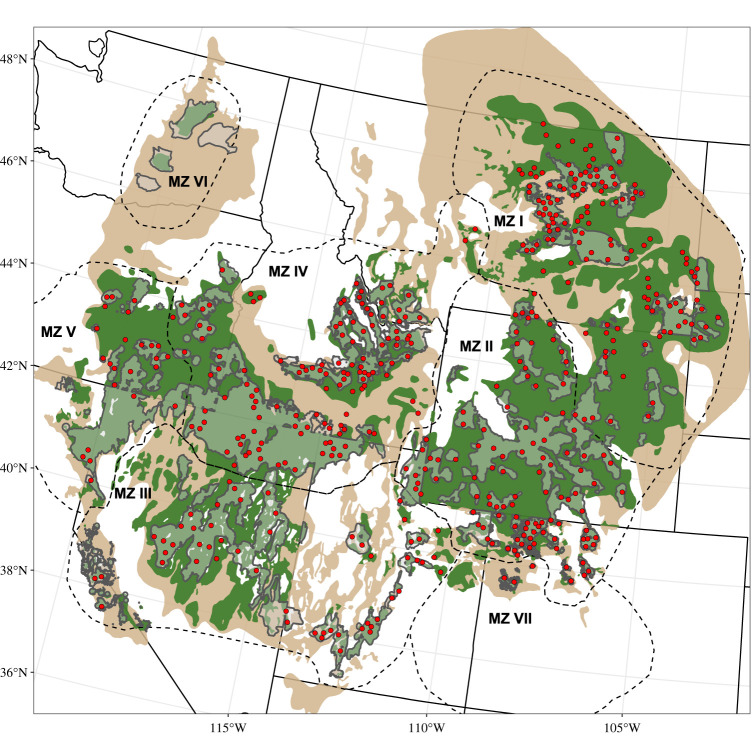
Greater sage-grouse priority areas for conservation (PACs; unbroken grey outlined polygons) delineated to alleviate threats to sage-grouse and their habitats, nodes among which connectivity was modelled (red dots), management zones (dashed black outlined polygons labelled MZs I–VII)—delineated using floristic zones [28]—within which functional connectivity was modelled (MZs I–V) [8], the species' contemporary (solid green polygons) and historical (pre-western settlement; solid tan polygons) range, and the western United States (solid black lines).

Sage-grouse management efforts have been anchored by billions of US dollars invested largely within priority areas for conservation (PACs) [27] (figure 1) alleviating threats to habitat and connectivity including cultivation, woodland expansion, infrastructure development and exotic annual grasses [29–32]. The 3.2 million ha PAC-based strategy was largely based on lek locations, while also including critical seasonal habitats [27] in an effort to protect breeding density, a design that encompasses 80% of breeding sage-grouse across approximately 25–34% of their range [33]. However, the PAC strategy was not designed to incorporate connectivity of populations [34]. Therefore, the concern among conservation partners is that well-intentioned PAC-based investments may not stave off population isolation as interstitial habitats degrade. Unknown is where within and among core areas practitioners might target efforts to protect, expand and connect the current PAC-based strategy.

Two recent advances in sage-grouse conservation genetics include a range-wide functional connectivity model (FCM) [8] and a network connectivity model (NCM) [11]. Models of these types (including these particular models) can and have been leveraged by conservation practitioners to identify landscapes that foster concentrated genetic exchange (FCM) and to identify and rank populations essential to maintaining range-wide genetic connectivity by network centrality (NCM). The NCM was developed to identify underlying pattern and strength of connectivity among leks, where genetic samples were collected, thereby identifying leks and connections among leks with greater and lesser importance to maintaining connectivity [11]. The NCM can be scaled to whatever geospatial scale is of interest; however, while leks are spatially referenced points on landscapes, the prioritized connections thereamong are inherently aspatial because movement pathways through interstitial habitats are unknown (i.e. not geospatially relatable nor landscape mechanism relatable). The FCM was developed to identify the process linking genetic structure to landscape mechanisms (e.g. terrain and land cover) known to influence sage-grouse connectivity, thereby identifying where connectivity is expected to be greatest and most concentrated [8]. The FCM provides a scalable map, lending itself to conservation design; however, while it does account for the highly heterogeneous nature of observed connectivity, it is not able to be prioritized with regard to conserving gene flow along pathways essential to maintaining the network of range-wide connectivity. When combined, the FCM and NCM can complement one another, enabling identification of the network of pathways among populations and prioritization by importance to range-wide genetic connectivity, prioritization that is geospatially scalable to landscape mechanisms and the management thereof.

Our objective was to synthesize these two models into one product that could provide practitioners a toolbox to prioritize individually ranked benefits of functional connectivity to populations. Once produced, we sought to investigate the extent to which PACs encompass connectivity and to leverage the product in three ways with the expressed intent of ingraining genetic connectivity more deeply into the PAC-based model: (i) identify landscapes vital to maintaining connectivity within PACs, (ii) identify PAC growth areas—those areas adjacent to PACs that might also be considered for protection—and pathways vital to maintaining connectivity among PACs and (iii) develop spatial action maps for mitigating impedances to connectivity.

## 2. Methods

### 2.1. Foundational models and connectivity map generation

We integrated two spatial models predictive of range-wide sage-grouse genetic connectivity: (1) a NCM; FCM [8] and (2) a genetic NCM [11].

(1) The FCM encompassed five of the seven sage-grouse management zones (MZs: delineations used for federal-level planning based on similar underlying environmental attributes [28]; figure 1). The MZs excluded were the two smallest and most isolated which comprise less than 1% of the sage-grouse range [35]. The FCM was developed and validated using genetic data and consisted of a resistance surface for each MZ using maximum-likelihood population-effects models to determine the effect of breeding habitat metrics, landscape attributes and indices of grouse abundance on genetic differentiation. Functional connectivity was reduced where the probability of occurrence for breeding leks was less than 0.25 or less than 0.5 and where the landscape was steeper with rougher terrain. Landscape attributes that reduced functional connectivity varied across MZs, and included sagebrush availability (less than 10–30%; MZs II, IV, V), tree canopy cover (greater than 10%; MZs I, II, IV), cultivation (greater than 25% in MZs I, II, IV; greater than 5% in MZ V) and human disturbance (greater than 0.09% in MZs I, IV, V). A composite model of range-wide connectivity was developed using Circuitscape [36] from the composite of the five individual MZ resistance surfaces, ranging in values from 1 (least resistance to gene flow) to 161 (greatest resistance to gene flow) at a resolution of 1200 m.

(2) The NCM, developed across the same sampling frame as the FCM, was based on a network analysis of gene flow resulting in the identification of 458 nodes (hierarchically clustered leks) and 14 433 edges connecting these nodes (representative of gene flow), and the relative importance of those edges to maintaining range-wide genetic connectivity [11].

Inherent strengths and weaknesses for conservation application varied by model (table 1). When combined, these models complement one another, enabling identification of the network of pathways among populations and prioritization by importance to range-wide genetic connectivity, prioritization that is geospatially scalable to landscape mechanisms and the management thereof.

**Table 1.** The connectivity designs possible when using each of the two foundational models—the functional connectivity model (FCM) [8] and the network connectivity model (NCM) [11]—and when using the product of integrating the two into a network prioritized functional connectivity model. When combined, the FCM and the NCM identify genetically connected nodes and the pathways among these nodes, enabling the prioritization of geospatially scalable pathways for the conservation of genetic connectivity and the understanding of the habitat drivers of said connectivity.

model	foundational model		product
	network connectivity model	functional connectivity model	network prioritized functional connectivity model
prioritizable^a^	☑	☒	☑
geospatially scalable^b^	☑	☑	☑
geospatially relatable^c^	☒	☑	☑
landscape mechanism relatable^d^	☒	☑	☑

^a^Can be used to determine conservation import of model entities (nodes/cells) relative to one another.

^b^Can be scaled to smaller spatial extents while retaining relevance to the full scale of the original model.

^c^The connections identified within the model are tied to geospatial pathways.

^d^The model identifies the landscape mechanisms underlying connectivity patterns.

To synthesize the two models, we restricted modelled connectivity to lek clusters identified as connected within the NCM and let the FCM serve as the empirical basis for resistance to gene flow. Thus, we modelled genetic connectivity for 14 433 pairwise NCM connections (edges) among 458 lek clusters (‘nodes’; figure 1) using circuit theory-based analysis of the FCM. The circuit theory-based approach treats the landscape as a circuit board which provides resistance to electrical flow among sources and grounds. In our analysis, the sources and grounds were the node pairs connected by edges in the NCM, current the gene flow thereamong and the circuit board the FCM resistance surface raster whose values indicate landscape resistance to gene flow [37]. We set Circuitscape parameters to pairwise mode and considered movement from each raster cell to all eight neighbouring raster cells (Queen's case). Though computationally intensive [38], this approach outperforms more simplistic approaches which tie movement to a single optimal route and which assume perfect knowledge of the landscape [7,39–41]. We output pairwise resistance values and current maps. Resistance values represent the total resistance encountered along the path of least resistance among nodes, and current maps demonstrated the magnitude of functional connectivity for each raster cell (henceforth referred to as connectivity maps), and when all summed resulted in a cumulative functional connectivity map (henceforth referred to as the cumulative connectivity map).

### 2.2. Pathway prioritization and delineation

First, we identified areas with the greatest benefit to cumulative connectivity by thresholding the cumulative connectivity map from the 50th to 95th percentile incremented by 5%, including the 99th percentile. This approach provided a means to identify and prioritize landscapes that contributed the greatest connectivity for the greatest number of connections. Impedances to connectivity constrain connectivity, and cumulative connectivity behaves predictably by coalescing into visible pathways with increasing thresholds (e.g. the 90th, 95th and 99th percentile). We selected a 5% threshold to show the utility of delineating pathways into spatial action maps though thresholds can be scaled for ranking range-wide or within-state priorities.

Second, we prioritized pairwise pathways among nodes using a two-step process. (i) We ranked the NCM minimum spanning tree (MST) edges by betweenness. (ii) We delineated pathways corresponding to these prioritized edges by thresholding each edge's corresponding connectivity map to the 99th, 99.5th and 99.75th percentiles. Within the NCM, the MST is the subset of edges connecting all nodes, representing maximum genetic covariance, without a node being connected back to itself. Edge betweenness is an index of the number of pairwise connections among nodes fostered by a given edge in the NCM; larger values represent greater genetic interconnectivity [42,43]. This approach provided a means to identify and prioritize individual pairwise corridors based on their contribution to range-wide genetic connectivity.

### 2.3. Cumulative connectivity within and among priority areas for conservation

Targeted management within PACs remains a priority, while ensuring connectivity among PACs provides direction to growing core habitats for population connectivity. Thus, we subset the thresholded cumulative connectivity map to landscapes each within PACs and among PACs to identify key connectivity core and growth opportunities. We evaluated how well connectivity was enveloped by PACs by calculating at each decile (10% quantile) of cumulative connectivity the proportion within and outside of PACs.

### 2.4. Impedances to connectivity

Previous work demonstrated that thresholds exist in cultivation and tree canopy cover which, when exceeded, inhibit functional connectivity [8]. We mapped where cultivation and woodland expansion exceeded identified thresholds; specifically, where cultivation was greater than 25% within MZs I, II, IV and V, and where woodland canopy cover exceeded 10% within MZs I, II and IV (figure 1 and electronic supplementary material; both calculated within a 6.44 km radius moving window, and then resampled at 1200 m resolution for analyses to match [8]). We summed the areas exceeding thresholds within the top 5% of cumulative connectivity (the 95th percentile) inside and the top 5% of cumulative connectivity outside of PACs. We used contemporary (2020) imagery for both cultivation [44] and woodland cover [45] to identify current impedances to sage-grouse connectivity.

### 2.5. Software and spatial data

Data processing, analyses and post-processing were performed in R [46]—using packages *ggplot2* [47], *maps* [48], *raster* [49], *scales* [50], *sf* [51] and *sp* [52,53], Circuitscape implemented within Julia [38,54], and Google Earth Engine [55]. Additional map data were sourced and are publicly archived and available as follows: MZs [28], PACs [56], historic greater sage-grouse range [57] and contemporary greater sage-grouse range [58].

## 3. Results

### 3.1. Foundational models and connectivity map generation

**Figure 2.**
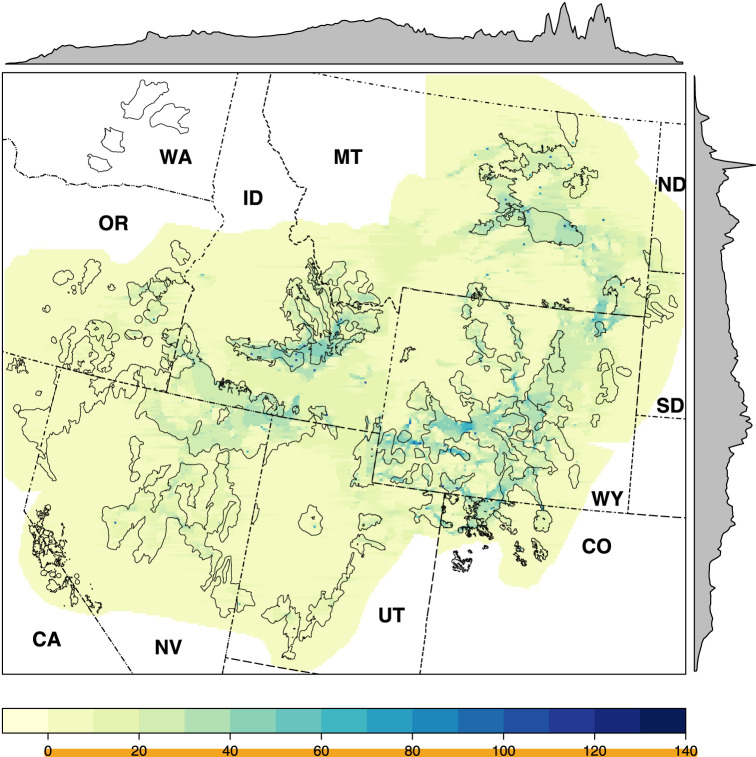
Sage-grouse cumulative connectivity across the sampled greater sage-grouse range in the western United States (dash-dotted lines and two-letter abbreviations). Cumulative connectivity is the sum of all pairwise connections (ramped from low (0, yellow) to high (140, dark blue)). Priority areas for conservation (PACs; solid grey lined polygons)—largely developed using breeding density—encompassed approximately twice as much cumulative gene flow when compared to randomly delineated equal area bootstrap spatial samples, though areas with high concentrations of gene flow occur outside PACs. Margin plots depict the relative magnitude of the median cumulative connectivity along the two primary axes.

Across much of the sage-grouse range, functional connectivity was relatively uninhibited (FCM resistance surface: range = 1–161, median = 10) as manifested in the pairwise resistance values resulting from the circuit theory analysis thereof (range = 0.51–16.58, median = 3.52; electronic supplementary material, table S1 and figure S1), though a small number of connections held exceptional importance to network-wide connectivity (NCM edge betweenness: range = 1–316, median = 8; electronic supplementary material, table S2 and figure S2). Cumulative connectivity ranged widely (range = 0.0017–121.85, median = 6.59; electronic supplementary material, table S1 and figure S1). The largest, contiguous landscapes with the greatest concentration of connectivity were largely central to the species' range (figure 2: across ID, MT and WY). Many smaller tracts important to maintaining connectivity among peripheral populations were speckled across the range, and there exist many areas of dispersed cumulative connectivity (figure 2) especially among peripheral populations.

### 3.2. Pathway delineation, prioritization and cumulative connectivity within and among priority areas for conservation

**Figure 3.**
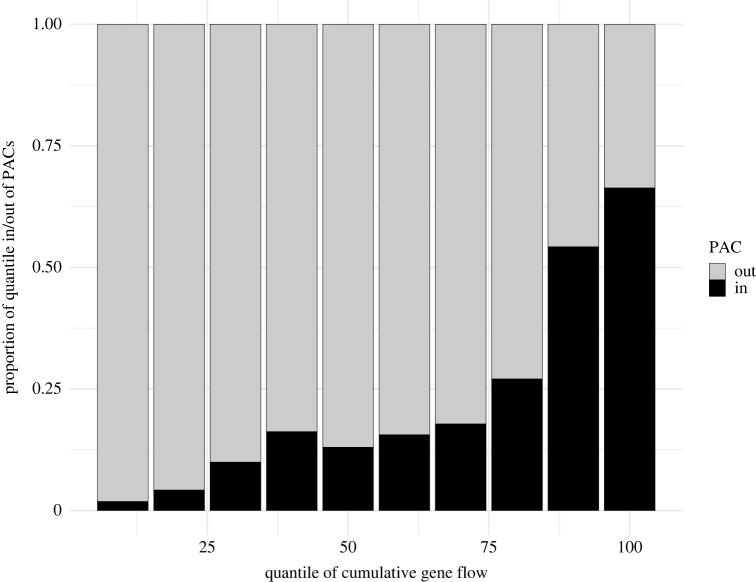
Proportion of cumulative gene flow encompassed by (in) or excluded from (out) sage-grouse priority areas for conservation (PACs). Results are depicted by increasing 10% deciles of cumulative gene flow. PACs encompass 41.1% of the total connectivity, and contain greater than 54% of cumulative connectivity among the top 20th percentile of all values.

**Figure 4.**
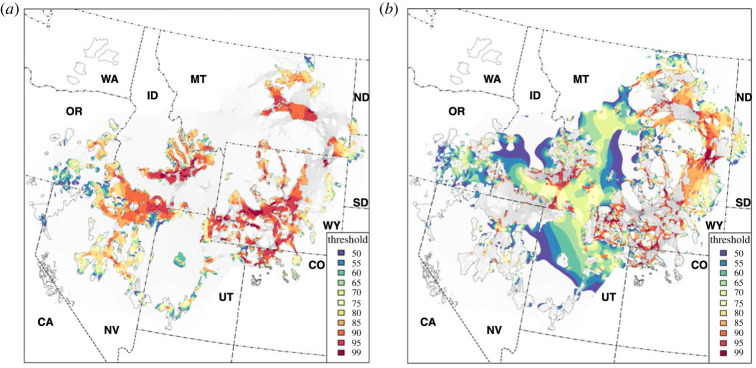
Priority cumulative pathways for connectivity in (*a*) and out (*b*) of greater sage-grouse priority areas for conservation (solid black line polygons) [27] in the western United States (dash-dotted lines and two-letter abbreviations), thresholded percentile (spectral colours) of cumulative connectivity (underlying image: low (light grey) → high (dark grey)). Cumulative connectivity was calculated among all 458 connected nodes following the network connectivity model of Cross *et al*. [11] based on the functional connectivity model resistance surface of Row *et al*. [8].

**Figure 5.**
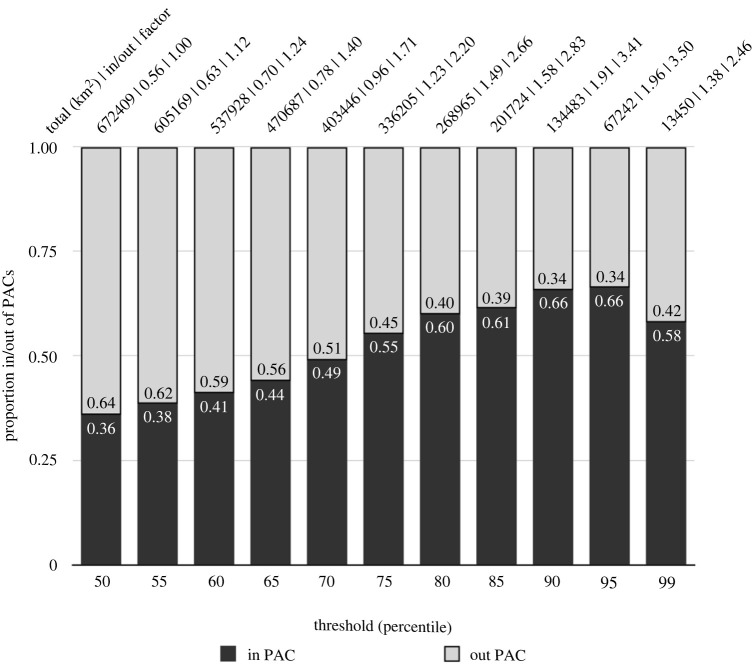
Area-based results observed from different threshold values applied to the cumulative connectivity map. The cumulative area (total (km^2^)) considered, the proportion in and out of sage-grouse priority areas for conservation (PACs) (labels below/above split in bars), with the ratio (in/out) between the two values, and the factor of this ratio relative to the most inclusive threshold considered (50th percentile) providing an index of the relative value within areas currently targeted for conservation.

Sage-grouse PACs encompassed 41.1% of cumulative connectivity, disproportionately encompassing landscapes with the highest cumulative connectivity: greater than 50% of the top 20% of cumulative connectivity (80th percentile) was enveloped within PACs (figure 3). The relationship between the per cent total area thresholded within PACs (figure 4*a*) divided by that among PACs (figure 4*b*) increased steadily to a maximum observed factor of 3.5 at the 95th percentile threshold (figure 5). A 70th percentile threshold yielded an area inside PACs nearly equivalent to the area outside PACs. Individual pathway overlap with PACs varied widely; some pathways were almost completely inside PACs (97.2% overlap) while others were barely so (4.7%; mean = 62.0%).

Cumulative connectivity was greatest within the largest contiguous area PACs (figure 4*a*), especially those more central to the species’ range (e.g. central WY), but was occasionally peripheral where landscapes fostered connectivity among neighbouring PACs (e.g. ID and central MT). The greatest cumulative connectivity among PACs included both pathways connecting and additions immediately adjacent to PACs (figure 4*b*). Thresholding cumulative connectivity to the 65th percentile resulted in continuous connectivity between PACs in the eastern (the states of CO, MT, ND, SD, WY) and western (CA, ID, NV, OR, UT) halves of the species' range. At thresholds of greater than the 90th percentile, connectivity among many peripheral PACs was achieved (e.g. within parts of OR, NV and CO) ensuring connectivity among all neighbouring states.

**Figure 6.**
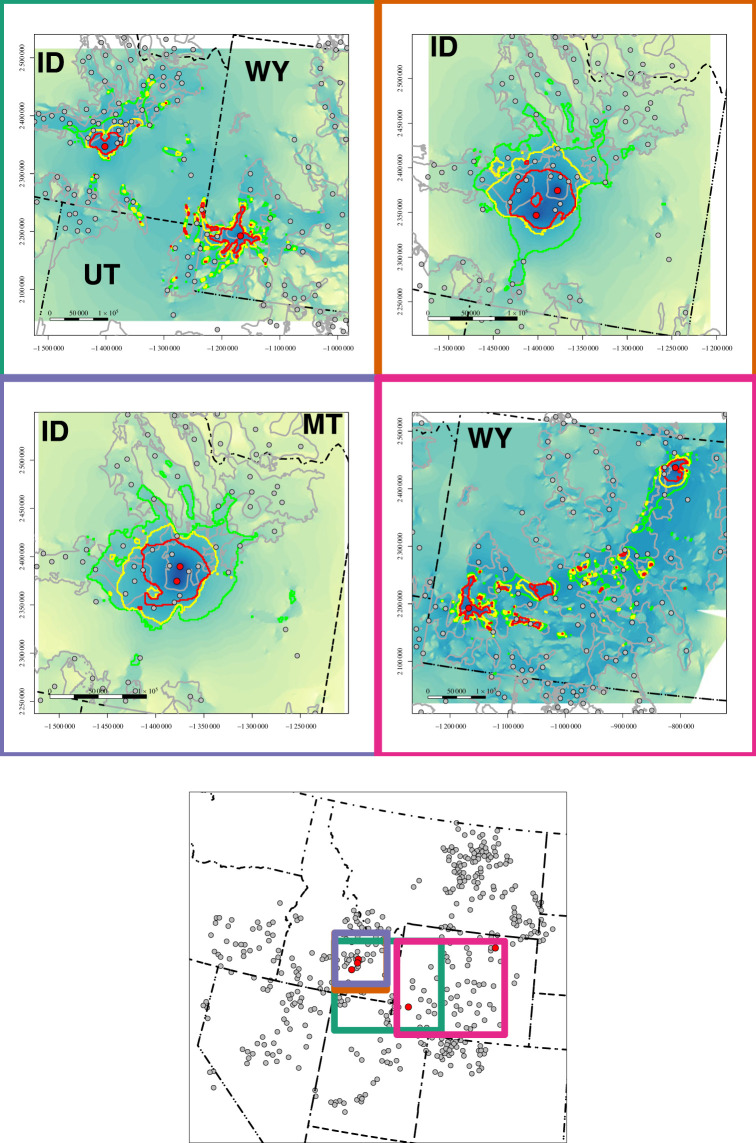
Pathways delineated from the top 1% (green polygon), top 0.05% (yellow polygon) and top 0.025% (red polygon) of greater sage-grouse pairwise functional connectivity between the top four network model minimum spanning tree pairwise connections (as ranked by betweenness: node 99 to node 545 = 51 600 (green border), 61 to 99 = 51 480 (orange border), 57 to 61 = 49 737 (purple border), 545 to 74 = 46 041 (magenta border)). Also shown are the pair of nodes connected (red dots) and other nodes in the vicinity (grey dots), greater sage-grouse priority areas for conservation boundaries (grey polygons), western United States borders (black dash-dotted lines), and the underlying pairwise connectivity map (low: yellow → high: blue). Inset map shows extents of maps and all 458 nodes (grey dots). Top node pairs were 280.4 km (node 99 to node 545), 36.6 km (61 to 99), 15.5 km (57 to 61), 434.1 km (545 to 74) apart (left to right, top to bottom). Coordinates are in metres projected in Albers Equal Area Conic USGS.

The top four pairwise pathways among nodes prioritized by NCM MST edge betweenness and delineated by thresholded pairwise connectivity were located central to the species’ range and involved three high betweenness nodes (figure 6). These pathways connected leks in ID to leks in southwest WY, leks within ID and leks in southwest WY to leks in northeast WY (in order of priority). Increasing thresholds delineated smaller pathways, acting as buffers surrounding connected leks when connections were proximal and forming stepping-stone pathways when connections were distal. Decreasing thresholds to incorporate more pairwise connectivity increases pathway size and incorporates more pathways.

### 3.3. Impedances to movement

**Figure 7.**
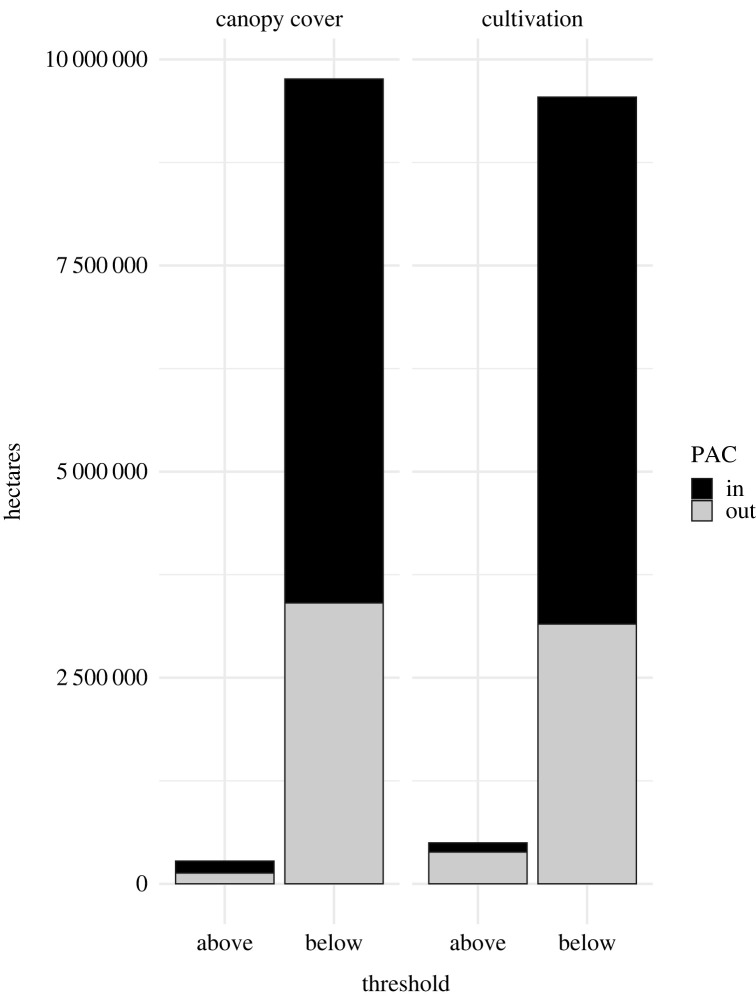
The total area (ha) within the top 5% (95th percentile) of cumulative connectivity above or below the woodland canopy cover and cultivation connectivity impedance thresholds identified by Row *et al*. [8]. Areas were calculated for both inside and outside of greater sage-grouse priority areas for conservation (PACs).

**Figure 8.**
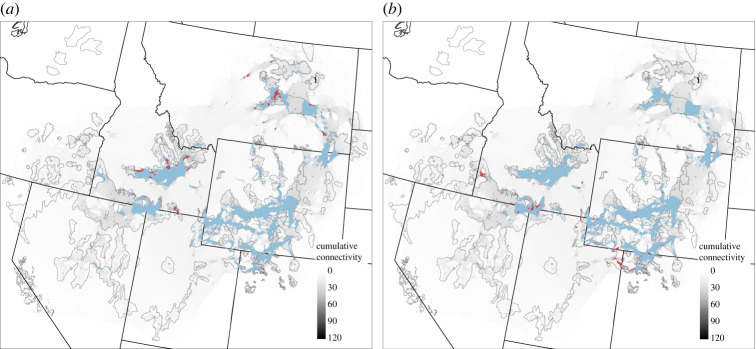
Areas in red identify landscapes exceeding previously identified thresholds in impedances to greater sage-grouse connectivity due to either cultivation (*a*) or woodland canopy cover (*b*). Thresholds were applied within the top 5% of cumulative connectivity (light blue) to provide practitioners with spatial action maps to abate impedances to connectivity within important areas for connectivity. Cumulative connectivity is the sum of all pairwise connections and is ramped from low (grey) to high (black). Greater sage-grouse priority areas for conservation (solid black line polygons) and the western United States (solid black lines) are also shown for reference.

Among landscapes containing the top 5% of cumulative connectivity (67 242 km^2^; figure 5), 3.8% (2555 km^2^) exceeded the cultivation threshold, 29% (741 km^2^) of which was within PACs (figure 7), with areas generally found in eastern MT and surrounding the Snake River Plain in ID (figure 8). Similarly, only 3.3% (2219 km^2^) exceeded woodland canopy cover thresholds (figure 7), though 57% (1265 km^2^) was within PACs (figure 8).

## 4. Discussion

Our network prioritized FCM integrates the combined strengths of functional connectivity and network analyses, enabling the creation of spatial action maps prioritized by importance to maintaining range-wide genetic connectivity. Previous approaches have identified key connectivity landscapes using a variety of approaches—e.g. smoothed cumulative least cost paths [59] or maximum landscape permeability [60]. Our approach is novel in that we identify and empirically prioritize pathways, enabling quantification of the relative contribution of each pathway to maintaining range-wide genetic connectivity. Prioritizations scale from local- to biome-level, allowing decision makers to maximize the collective return on investments in connectivity [61], and creating an opportunity to incorporate connectivity into sage-grouse conservation.

PAC-based population strongholds serve well to envelop genetic connectivity. However, highly targeted actions within and among PACs could conserve additional pathways, perpetuating range-wide conservation of the species. While much of the occupied range of sage-grouse exhibited low resistance to movement, suggesting numerous options for conserving connectivity, we have identified those landscapes that evidentially foster the greatest and most concentrated connectivity; landscapes located across ID, central MT and central WY and generally within the largest contiguous area PACs. Our maps of thresholded cumulative connectivity within and among PACs can help guide maintenance of pathways offering maximal benefit to connectivity.

Landscape composition and habitat quality within priority pathways are highly variable across the range. Within the 95th percentile of cumulative connectivity, the total area of landscapes exceeding the cultivation threshold is greater than that of those exceeding the woodland canopy threshold. However, within PACs, woodland canopy thresholds are exceeded over nearly 1.7 times that of cultivation. Overlaying cumulative connectivity maps with these previously identified impedances highlighted areas that require active restoration to restore connectivity capacity. However, more efficient strategies may be found in targeting at-risk landscapes for intervention before thresholds are exceeded. For example, conservation easements are one tool for alleviating the potential threat of expanding cultivation [62], and targeting early encroaching conifers for removal has demonstrated benefits to both breeding sage-grouse [63,64] and other sagebrush obligate species [65]. The treated acreage required to alleviate these impedances is likely within grasp: we identified 276 514 ha of areas exceeding canopy cover thresholds underlying the top 5% of cumulative connectivity, while over 280 000 ha of conifer removal has already taken place under the Sage Grouse Initiative alone [30,31]. There are almost certainly additional impedances to sage-grouse genetic connectivity including invasive annual grasses [66] and energy infrastructure [67]. Furthermore, our approach to creating spatial action maps can be used on landscapes forecast using climate models to determine future action areas. The addition of our cumulative connectivity map to the conservation portfolio for sage-grouse allows for explicit inclusion of these impedances into spatial action maps for connectivity.

Areas of concentrated connectivity identified via circuit theory may not equate to the highest quality habitat, but may instead be areas where movement is restricted by landscapes unsuitable to connectivity which, at the extreme, can present barriers. Therefore, pathways identified by our approach can be considered conservation priority to maintain connectivity, though lesser cumulative connectivity does not necessarily mean lesser conservation importance, especially when considering connectivity with peripheral populations often linked thereby. Again, our thresholds are not intended to represent recommended cut-offs so much as an example of how to delineate pathways from our cumulative connectivity map; therefore, practitioners interested in delineating pathways might consider thresholds commensurate with the area of the landscape between nodes able to be conserved. For example, geographically distal nodes may warrant a greater threshold to limit the total area considered for conservation.

The priority pathways we identified likely reflect the landscapes essential to maintaining functional connectivity up to and including the time of sample collection. For organisms capable of moving large distances, like sage-grouse, the lag time from the genesis or dematerialization of an impermeable barrier until that change is detectable in population allele frequencies could be 1–15 generations (less than or equal to 30–45 years) [68]. Therefore, contemporary landscape alterations may have already affected our priority pathways, such as where impedances to movement exceed thresholds. For example, the cultivation and woodland thresholds identified from contemporary imagery by Row *et al*. [8] may actually exceed the true threshold at which these impedances first began affecting connectivity. Therefore, where landscapes present and future exceed these thresholds identified, mitigation should still benefit sage-grouse. Finally, our identified pathways are in some cases discontinuous. Conservation of such landscapes for flightless terrestrial organisms may not be efficacious. However, the long-distance dispersal capability of sage-grouse [24,25] characterized by punctuated movements linking stepping-stone stopovers [26] suggests identified pathways could support genetic connectivity conservation.

In cases where functional connectivity cannot be maintained or has been lost due to irreversible landscape degradation, translocation of individuals may serve to re-establish historical populations, augment failing populations and provide genetic rescue to small, imperilled populations. However, translocations should be conducted judiciously as there exist the potential risks of outbreeding depression and swamping of local adaptation [69,70] as well as that of low initial survival rates of translocated individuals [14,71]. In our opinion, the efficacy of translocations is limited from an ecosystem perspective because they (i) only benefit a single species, (ii) do not directly address the root cause of population isolation and (iii) may not be biologically self-sustaining, whereas the prioritization of sagebrush connectivity resulting from conserving greater sage-grouse priority functional connectivity should (i) benefit multiple species, (ii) address the root causes that likely lead to population isolation and (iii) result in more self-sustaining population augmentation through the conservation of natural landscape functional connectivity.

Our model synthesis relied on a FCM developed using a k-folds cross-validation approach based on neutral genetic markers. Inference from such models can be confounded by the success of past translocation of individuals, which might result in the identification of inflated landscape connectivity. Thus, we urge careful consideration of our priority corridors among landscapes with a known history of translocations. In these cases, and in general, additional methods might be used to validate the pathways we identify. These could include the genetic identification of migrants and their offspring or the integration of direct measures of animal movement estimated from radio-marked individuals. Movement data could serve as a proxy to evaluate the efficacy of our model as applied to future conservation strategies. However, we note the majority of direct measures of animal movement do not quantify the functional (i.e. demographic) connectivity of populations. Furthermore, individual response to habitat structure will vary among species, so FCMs for other sagebrush-obligate species or those that dwell within the sagebrush ecosystem could provide insight into the generality of our priority corridors [8]. Finally, future work that integrates predictions of climate-driven landscape change across the sagebrush biome into a FCM should improve on our predictions of priority corridors.

Prioritizable and scalable pathways identified here provide a complement, rather than an overhaul, to a PAC-based conservation design. Since inception, a concern underlying PACs has been the possibility that continued degradation would render PACs isolated ‘zoos’ facing a multitude of threats to sagebrush-obligate populations [34]. Our findings indicate that the current conservation strategy maintains disproportionately large reservoirs of connectivity within PACs, and can be used to identify where conservation actions outside of PACs—specifically, pathways used to grow and connect PACs—might most benefit genetic connectivity as part of a proactive conservation strategy. Maintaining robust breeding populations and connectivity will be paramount in light of the anticipated changes to the sagebrush biome resulting from climate change [72,73]. Sage-grouse functional connectivity now represents a unique case for inclusion into conservation planning.

## 5. Conclusion

Models of species distribution and abundance often guide a ‘core areas’ approach to conserving wildlife populations by targeting management and protections around key seasonal habitats. Though these strategies often wholly omit genetic connectivity in designs, or rely on assumptions about genetic connectivity from movement data and species–habitat relationships. Our novel approach gives practitioners the framework to prioritize conservation beyond seasonal habitats with confidence that their actions can help maintain pathways of connectivity between priority landscapes. Beyond sage-grouse conservation, our method for prioritizing pathways for wildlife connectivity conservation is broadly applicable across taxa, where both functional and network connectivity model types exist or can be generated from genetic data. Furthermore, combining our approach with known threats can form the foundation for comprehensive conservation planning to complement more traditional reserve designs.

## Data accessibility

Data products and metadata are publicly available as follows. (i) FCM microsatellite and spatial data: https://doi.org/10.5066/F7RB73V0 [74]. (ii) NCM microsatellite, spatial and network attribute data: https://doi.org/10.5066/F73N22PN [75]. (iii) Cumulative connectivity map: https://doi.org/10.5066/P9HI7OGR [76]. Additional details on these foundational model datasets as well as the computer code required to conduct our analyses are documented within the electronic supplementary material [77].
